# Editorial: Mitochondrial plasticity and quality control in health and disease

**DOI:** 10.3389/fcell.2024.1468818

**Published:** 2024-08-02

**Authors:** Francesca Forini, Elena Levantini, Emilia Bramanti, Filippo Maria Santorelli, Azhar Ali, Vincenzo Lionetti, Milena Rizzo

**Affiliations:** ^1^ Institute of Clinical Physiology (IFC), National Research Council (CNR), Pisa, Italy; ^2^ Institute of Biomedical Technologies (ITB), National Research Council (CNR), Pisa, Italy; ^3^ Institute of Chemistry of Organometallic Compounds (ICCOM), National Research Council (CNR), Pisa, Italy; ^4^ Molecular Medicine & Neurobiology, IRCCS Fondazione Stella Maris, Pisa, Italy; ^5^ Cancer Science Institute Singapore, Singapore, Singapore; ^6^ Interdisciplinary Research Center “Health Science”, Scuola Superiore Sant’Anna, Pisa, Italy; ^7^ UOSVD Anesthesia and Resuscitation, Pisa, Italy

**Keywords:** mitochondria quality control, mitochondrial genome, mitochondrial disorders, mitochondrial dysfunction in pathologies, mitochondria in development and differentiation

## 1 Introduction

Mitochondria play a multifaceted role in cellular physiology extending from the regulation of energy production and cell metabolism to calcium buffering and the modulation of intracellular signaling. Given that mitochondrial dysfunctions are involved in a spectrum of complex human pathologies and inheritable disorders, preserving a functional mitochondrial network is crucial for health and disease status and it has become a focal point of innovative therapeutic strategies.

This Research Topic compiles articles focused on two primary areas of mitochondrial research: i) the investigation of mitochondrial dysfunction in various pathological conditions (including mitochondrial diseases) and the exploration of therapeutic interventions that target and/or exploit mitochondria; and ii) the elucidation of the crucial role of mitochondria in development and differentiation. Finally, this issue introduces a novel methodology that employs commercially available tools for the quantification of mitochondrial DNA and RNA within intact and complex tissues.

## 2 Mitochondrial disfunction in pathological conditions

### 2.1 Cardiovascular diseases

Preservation of mitochondrial homeostasis through mitochondrial quality control (MQC) is fundamental for organs with high energy demands, such as the heart. The comprehensive review by Atici et al. underscores the significance of MQC in the context of cardiovascular physiology and various disease states, including myocardial ischemia-reperfusion injury, atherosclerosis, heart failure (HF), cardiac hypertrophy (CH), hypertension, and both diabetic and genetic cardiomyopathies, as well as Kawasaki Disease. Enhancing mitochondrial function holds promise for mitigating tissue remodeling, preventing the onset of cardiovascular disease, regenerating the myocardium and potentially extending lifespan. Cardioprotection might be achieved through the application of either synthetic or natural mitochondria-targeted compounds that promote balanced mitochondrial fusion and fission, mitophagy, bioenergetics, ROS detoxification, replenishment of the mitochondria pool, and enhancement of inter-organelle communication.

Among the natural compounds, Moku-boi-to (MBT), a Japanese medical herbal concoction, has demonstrated promising protective effects in both *in vivo* and *in vitro* models of CH by restoring mitochondrial dynamics (Tagashira et al.). Altered mitochondrial fragmentation, which leads to excessive mitophagy and decreased ATP production, thus favoring ROS generation, can be addressed by MBT treatment (Quiles and Gustafsson, 2022; Yang et al., 2022). The authors demonstrated that in a mouse model of HF, MBT treatment improved heart contractility and mitigated CH (Tagashira et al.). Mechanistically, MBT prevents the accumulation of the mitochondrial pro-fission protein Dynamin 1 Like (DRP1) and the resultant mitochondrial fragmentation induced by hypertrophic stimuli, while concurrently reducing mitochondrial dysfunction, ROS production, intracellular Ca^2+^ dysregulation, and cell death.

The dynamic balance of mitochondrial fission and fusion ensures a prompt adaptation to the cell’s rapidly changing metabolic demands (Tilokani et al., 2018). Metabolic reprogramming, favoring glucose utilization over fatty acid oxidation, is a hallmark of both heart disease and malignant cell growth, shapes the epigenetic landscape of cardiomyocytes enabling heart regeneration (Li et al., 2023) and making mitochondria strategic targets in the cardio-oncology field (Karlstaedt et al., 2022). In a comprehensive review, Chen et al. provide insights into the complex processes that involve mitochondrial impairments in doxorubicin (DOX)-induced cardiotoxicity, with an emphasis on the metabolic consequences. Rebalancing mitochondrial dynamics by inhibiting DRP1-mediated mitochondrial fission and enhancing Mitofusin 2 (MFN2)-mediated mitochondrial fusion, could promote chromatin reconfiguration that is correlated to the shift in cellular energy metabolism from glycolysis to fatty acid oxidation with maintenance of oxidative phosphorylation. This dual-action has the potential to decrease the cardiac adverse effects of DOX, while enhancing its anti-cancer efficacy.

### 2.2 Metabolic diseases

In the skeletal muscle (SkM), a reduction in the elongated mitochondrial phenotype, indicative of dysregulated mitochondrial dynamics, is a feature of metabolic disorders such as type 2 diabetes mellitus (T2DM). Using SkM biopsies from T2DM patients, Castro-Sepulveda et al. showed that the activation of toll-like receptor 4 (TLR4), a mediator of both infectious and non-infectious inflammatory diseases (Wei et al., 2023), contributes to significant changes in mitochondrial morphology and cristae density. This effect is mediated by a reduction in the levels of the pro-fusion protein Mitochondrial Dynamin like GTPase (Opa1). Given the critical role of SkM in the pathophysiology of T2DM (DeFronzo, 2009), this mitochondrial-related signaling cascade may contribute to insulin resistance in T2DM thus exacerbating hyperglycemia-induced multiple organ failure.

Non-alcoholic fatty liver disease (NAFLD) represents another metabolic condition where mitochondrial remodeling plays a key role (Zheng et al., 2023). A recent observational case-control study highlighted that NAFLD patients (Houshmand et al.) present with an increased hepatic mitochondrial DNA copy number, a surrogate marker of mitochondrial functionality (Castellani et al., 2020). This finding correlated with increased expression of Fibroblast Growth Factor 21 (FGF21), a regulator of glucose and lipid metabolism, energy homeostasis, and insulin sensitivity (Cuevas-Ramos et al., 2009). These findings suggest an adaptive response aimed at increasing the liver’s capacity to metabolize fat. However, this compensatory response may ultimately turn detrimental by causing uncontrolled oxidative stress and progressive mitochondrial dysfunction.

### 2.3 Mitochondrial diseases

Mitochondrial dysfunctions are not only confined to complex-diseases; they can also arise from pathological variants in genes crucial for mitochondrial function and MQC. Given the genetic heterogeneity of mitochondrial disorders (MDs) and the current lack of effective therapeutic options, establishing a precise genetic diagnosis can significantly enhance our understanding of the disease. This, in turn, may lead to an improved ability to manage it.

The innovative use of next-generation sequencing (NGS) approaches, coupled with comprehensive analysis of the entire mitochondrial genome, as demonstrated by Nogueira et al., 2024 helped achieve genetic diagnoses across a wide demographic, encompassing both pediatric and adult cases. Notably, variants in mtDNA are predominantly associated with adult-onset MDs, while nuclear DNA variants are more frequently linked to early-onset forms of MDs.

### 2.4 Intercellular mitochondrial transfer in pathology and therapy

Mitochondrial transfer has been recently recognized as a crucial process for MCQ (Liu et al., 2023) and has emerged as a novel therapeutic approach in disease treatment (Clemente-Suárez et al., 2023). In their seminal review, Zhang et al., explored the role of mesenchymal stromal/stem cells (MSCs) in pulmonary development and their dysfunction’s contribution to bronchopulmonary dysplasia (BPD). The review focuses on the efficacy of MSC therapy in treating BPD, a condition linked to defects in mitochondrial structure, dynamics, and oxidative metabolism. MSC treatment has been shown to decrease mitochondrial dysfunction by i) stimulating mitochondrial replication in injured lung cells and by ii) restoring mitochondrial function through the direct transfer of healthy mitochondria from MSCs to the damaged primary cells of the lung. It is conceivable that tissue repair is mediated by mitochondria transferred from MSC-derived cells, similar to the observations obtained following transplantation of mitochondria-rich extracellular vesicles in the heart (Ikeda et al., 2021).

However, mitochondrial transfer can promote adverse effects favoring pathological conditions. For instance, Cereceda et al. suggested that the uptake of platelet-derived mitochondria by triple-negative breast cancer cell lines increases mitochondrial respiration, ATP production, cell proliferation, and metabolic adaptability. This enhancement in cellular functions may contribute to tumor progression, underscoring the multifaceted nature of mitochondrial transfer in the context of diseases.

## 3 Mitochondrial function in differentiation and development

Myogenic differentiation, a key step in skeletal muscle regeneration, is characterized by significant changes in both the quantity and functionality of mitochondria to meet the increased energy demand. Throughout this process, transient and mild cellular stress triggers a variety of mitochondrial response pathways, collectively termed mitochondrial stress responses (MSRs), which favor cellular homeostasis. The review by Lin et al., described MSRs and their involvement in the regulation of physiological myogenic differentiation, providing new perspectives into potential treatments for muscle diseases associated to impaired myogenic differentiation.

Superoxide dismutase 2 (Sod2) is a key mitochondrial enzyme involved in MSR, which plays a primary role in ROS detoxification. Tarbashevich et al., using zebrafish models, demonstrated that Sod2 is also involved in the “purifying selection” of primordial germ cells (PGMs). Such process safeguards mitochondrial integrity across generations, ensuring the propagation of mtDNA devoid of deleterious mutations (Schwartz et al., 2022). Results by Tarbashevich et al. suggest that PGCs deficient in Sod2 are less competitive in generating gametes compared to their wild-type counterparts, due to the selective disadvantage imposed by higher levels of ROS-induced damage.

## 4 Genome quantitation

Finally, Giarmarco et al. introduced a new method for the spatial quantification of both mtDNA and mtRNA within fixed frozen tissue sections. This method employs a dual-probe strategy that combines RNAscope™ *in situ* hybridization (ISH) with immunohistochemistry (IHC) for precise labeling of mitochondria. Such a technique could prove to accurately quantify mtDNA copy numbers and expression levels, while preserving the integrity of cell morphology within its native microenvironment. The authors applied this methodology in a complex tissue like the retina, with the aim to evaluate circadian variations in mtDNA and mtRNA levels in zebrafish cone and rod photoreceptors, at an individual cells’ resolution.

## 5 Conclusion

Mitochondria are metabolic hubs, essential for cellular homeostasis, whose dysregulation is associated with a broad range of diseases. Targeting mitochondria is therefore an attractive strategy, and precision medicine based on mitochondria is emerging as a rapidly evolving field. The multi-layered research presented in this Research Topic emphasizes the crucial impact of this organelle in both health and disease. The studies highlight our increased understanding of mitochondrial dynamics within their cellular milieu and suggest the feasibility of targeting mitochondrial dysfunctions as a viable strategy for a range of pathologies, from cardiovascular diseases to metabolic disorders. Additionally, advancements in genetic diagnostics promise to refine our ability to manage mitochondrial disorders more effectively. Last, the exploration of mitochondrial transfer as a therapeutic intervention will require careful consideration of its dichotomous effects, offering benefits in regenerative medicine while posing challenges in cancer treatment. We anticipate a future where mitochondrial parameters emerge as pivotal biomarkers and therapeutic targets. The insights gained from these studies will inform the next-generation of mitochondrial researchers, propelling us towards more precise interventions.

## References

[B1] Castellani C. A. Longchamps R. J. Sun J. Guallar E. Arking D. E. (2020). Thinking outside the nucleus: mitochondrial DNA copy number in health and disease. Mitochondrion 53, 214–223. 10.1016/j.mito.2020.06.004 32544465 PMC7375936

[B2] Clemente-Suárez V. J. Martín-Rodríguez A. Yáñez-Sepúlveda R. Tornero-Aguilera J. F. (2023). Mitochondrial transfer as a novel therapeutic approach in disease diagnosis and treatment. Int. J. Mol. Sci. 24, 8848. 10.3390/ijms24108848 37240194 PMC10218908

[B3] Cuevas-Ramos D. Almeda-Valdes P. Aguilar-Salinas C. Cuevas-Ramos G. Cuevas-Sosa A. Gomez-Perez F. (2009). The role of Fibroblast growth factor 21 (FGF21) on energy balance, glucose and lipid metabolism. Curr. Diabetes Rev. 5, 216–220. 10.2174/157339909789804396 19531026

[B4] DeFronzo R. A. (2009). Banting Lecture. From the triumvirate to the ominous octet: a new paradigm for the treatment of type 2 diabetes mellitus. Diabetes 58, 773–795. 10.2337/db09-9028 19336687 PMC2661582

[B5] Ikeda G. Santoso M. R. Tada Y. Li A. M. Vaskova E. Jung J.-H. (2021). Mitochondria-rich extracellular vesicles from autologous stem cell-derived cardiomyocytes restore energetics of ischemic myocardium. J. Am. Coll. Cardiol. 77, 1073–1088. 10.1016/j.jacc.2020.12.060 33632482 PMC8626617

[B6] Karlstaedt A. Moslehi J. de Boer R. A. (2022). Cardio-onco-metabolism: metabolic remodelling in cardiovascular disease and cancer. Nat. Rev. Cardiol. 19, 414–425. 10.1038/s41569-022-00698-6 35440740 PMC10112835

[B7] Li X. Wu F. Günther S. Looso M. Kuenne C. Zhang T. (2023). Inhibition of fatty acid oxidation enables heart regeneration in adult mice. Nature 622, 619–626. 10.1038/s41586-023-06585-5 37758950 PMC10584682

[B8] Liu Y. Fu T. Li G. Li B. Luo G. Li N. (2023). Mitochondrial transfer between cell crosstalk - an emerging role in mitochondrial quality control. Ageing Res. Rev. 91, 102038. 10.1016/j.arr.2023.102038 37625463

[B9] Nogueira C. Pereira C. Silva L. Laranjeira M. Lopes A. Neiva R. (2024). The genetic landscape of mitochondrial diseases in the next-generation sequencing era: a Portuguese cohort study. Front. Cell Dev. Biol. 12, 1331351. 10.3389/fcell.2024.1331351 38465286 PMC10920333

[B10] Quiles J. M. Gustafsson Å. B. (2022). The role of mitochondrial fission in cardiovascular health and disease. Nat. Rev. Cardiol. 19, 723–736. 10.1038/s41569-022-00703-y 35523864 PMC10584015

[B11] Schwartz A. Z. A. Tsyba N. Abdu Y. Patel M. R. Nance J. (2022). Independent regulation of mitochondrial DNA quantity and quality in *Caenorhabditis elegans* primordial germ cells. Elife 11, e80396. 10.7554/eLife.80396 36200990 PMC9536838

[B12] Tilokani L. Nagashima S. Paupe V. Prudent J. (2018). Mitochondrial dynamics: overview of molecular mechanisms. Essays Biochem. 62, 341–360. 10.1042/EBC20170104 30030364 PMC6056715

[B13] Wei J. Zhang Y. Li H. Wang F. Yao S. (2023). Toll-like receptor 4: a potential therapeutic target for multiple human diseases. Biomed. Pharmacother. 166, 115338. 10.1016/j.biopha.2023.115338 37595428

[B14] Yang D. Liu H.-Q. Liu F.-Y. Guo Z. An P. Wang M.-Y. (2022). Mitochondria in pathological cardiac hypertrophy research and therapy. Front. Cardiovasc Med. 8, 822969. 10.3389/fcvm.2021.822969 35118147 PMC8804293

[B15] Zheng Y. Wang S. Wu J. Wang Y. (2023). Mitochondrial metabolic dysfunction and non-alcoholic fatty liver disease: new insights from pathogenic mechanisms to clinically targeted therapy. J. Transl. Med. 21, 510. 10.1186/s12967-023-04367-1 37507803 PMC10375703

